# Nontuberculous mycobacterial infection of the knee after arthrocentesis for idiopathic hemarthrosis: A case report

**DOI:** 10.1016/j.amsu.2021.102332

**Published:** 2021-04-16

**Authors:** Akihiro Moritake, Shigeshi Mori, Masato Kamiya, Kenji Yamazaki, Shingo Aoyama, Masao Akagi, Daisuke Togawa

**Affiliations:** a Departments of Orthopaedics and Rheumatology, Kindai University Nara Hospital, 1248-1 Otodacho, Ikoma, Nara, 630-0293, Japan; b Department of Orthopaedic Surgery, Kindai University Faculty of Medicine, 377-2 Ohnohigashi, Osakasayama, Osaka, 589-8511, Japan

**Keywords:** Nontuberculous mycobacterial infections, *Mycobacterium intracellulare*, Idiopathic hemarthrosis, Knee septic arthritis, Case report

## Abstract

**Introduction:**

Nontuberculous mycobacterial (NTM) infections of the musculoskeletal system are uncommon. Such infections are typically acquired by direct inoculation after penetrating trauma, surgical procedures, or needle injections. There are no reported cases of NTM infection after arthrocentesis for idiopathic hemarthrosis of the knee. Here we report a case of NTM infection in the knee that developed after arthrocentesis for idiopathic hemarthrosis of the knee.

**Presentation of case:**

The patient was an 85-year-old woman who experienced swelling of the left knee. An arthrocentesis was carried out, and hemarthrosis was found. The patient was referred to our hospital for repeated recurrence of hematoma of the knee. Significant swelling was observed in the suprapatellar sac. Magnetic resonance imaging examination revealed a mass at the suprapatellar sac. Laboratory data showed elevation of inflammatory markers. Debridement was performed under arthroscopy and samples were collected for culture. Although routine microbiological cultures were negative, the patient continued to experience knee swelling and laboratory data showed high C-reactive protein levels. Therefore, open debridement was carried out. At 4 weeks after the first surgical treatment, intraoperative cultures yielded *Mycobacterium intracellulare*. At this point, we diagnosed septic arthritis of the knee due to NTM infection. The patient showed an excellent prognosis with three-drug medical treatment for 1 year.

**Conclusion:**

Clinically, diagnosis of septic arthritis due to NTM infection is not easy. Because selection of examination depends on clinical suspicion, NTM infections should be considered for patients with elevation of inflammatory markers after episodes of surgical procedures, and/or needle injections.

## Highlights

• ・Nontuberculous mycobacterial (NTM) infections of the musculoskeletal system are uncommon.
• ・A case of NTM infection in the knee that developed after arthrocentesis.
• ・NTM infections should be considered for patients with elevation of inflammatory markers after needle injections.

## 1 Introduction

Nontuberculous mycobacterial (NTM) infections of the musculoskeletal system are uncommon. About 90% of NTM infections involve the pulmonary system, while the remainder involves lymph nodes, skin, soft tissues, and bones. The exact frequency of lower limb NTM infections is unknown [1]. NTM species are ubiquitously present in soil, tap water, animals, and other materials [2]. In the musculoskeletal system, NTM infections are typically acquired by direct inoculation after penetrating trauma, surgical procedures, and/or needle injections [3]. Previously, occurrence of arthritis due to NTM infection after intra-articular injection was reported [4]. However, NTM infection of the knee is an unusual entity, and there are no reported cases of NTM infection after arthrocentesis for idiopathic hemarthrosis of the knee. Here we report a case of NTM infection in the knee that developed after arthrocentesis for idiopathic hemarthrosis.

This article describes a single case reported in line with the SCARE criteria [5].

## 2 Presentation of Case

The patient was an 85-year-old woman who experienced pain and swelling in her left knee and visited a nearby hospital. The patient had ballottement of the patella in the left knee and underwent an arthrocentesis. The arthrocentesis revealed the presence of hemarthrosis. Although the symptoms improved after the procedure, the patient was subsequently referred to our hospital for repeated recurrence of hematoma of the knee. The patient had no significant past medical history, family history and no allergies.

**Fig. 1:**
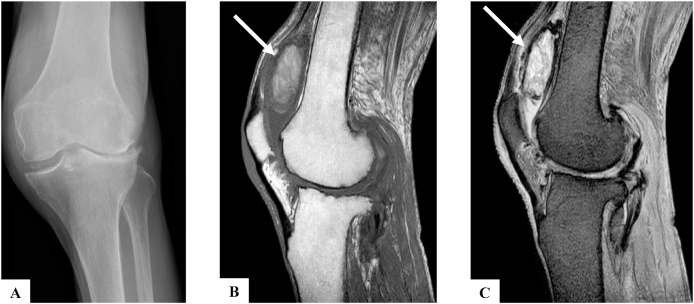
X-ray and MRI findings. A) X-ray showing lateral knee osteoarthritis. B) T1-weighted MRI image showing a hypointense appearance mass in the suprapatellar sac (arrow). C) T2-weighted MRI image showing a hyperintense appearance mass in the suprapatellar sac (arrow).

At the first visit to our hospital, there was ballottement of the patella and the knee range of motion was −30° of extension and 90° of flexion. Significant swelling was observed in the suprapatellar sac, but puncture was difficult because of the organization of the hematoma. Radiograph showed lateral knee osteoarthritis (Fig. 1A). Magnetic resonance imaging (MRI) examination showed a mass at the suprapatellar sac with a hypointense appearance on a T1-weighted image (Fig. 1B) and a hyperintense appearance on a T2-weighted image (Fig. 1C). Laboratory blood examination showed elevation of inflammatory markers: C-reactive protein (CRP) = 1.97 mg/dL; white blood cell count = 7500/μL (neutrophils, 75.4%; lymphocytes, 14.7%; monocytes, 8.4%; eosinophils, 0.7%; basophils, 0.9%); erythrocyte sedimentation rate (ESR) = 79 mm/h.

**Fig. 2:**
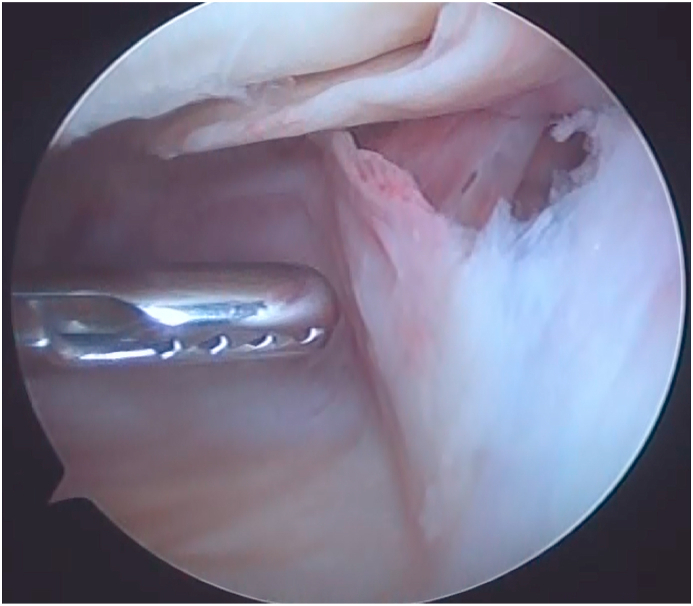
Arthroscopic findings. Only clots were found in the suprapatellar sac. Debridement was performed and samples were collected for culture and biopsy examinations.

Based on the MRI findings and medical history, we suspected that the mass was a hematoma, but infectious disease could not be denied because of the elevated inflammatory markers. Therefore, a diagnostic arthroscopy was performed. Two anterior portals were used in the surgery. Clots were observed, but no active bleeding was observed. We performed a debridement under arthroscopy and collected samples for culture and biopsy examinations (Fig. 2). Postoperatively, an antibiotic (cefazolin 1 g) was administered every 6 hours up to 18 hours.

From 10 days after the arthroscopy, the patient continued to experience knee pain and swelling, although routine microbiological cultures were negative. Laboratory data showed high CRP (11.29 mg/dL), ESR (>100 mm/h), and leucocytes at 9140/μL. Pathological examination showed only a fibrin mass, with no epithelioid cell granuloma or casement necrosis. Although the cultures were negative, we suspected septic arthritis arising from an unknown causative organism that would become worse and performed an open debridement.

**Fig. 3:**
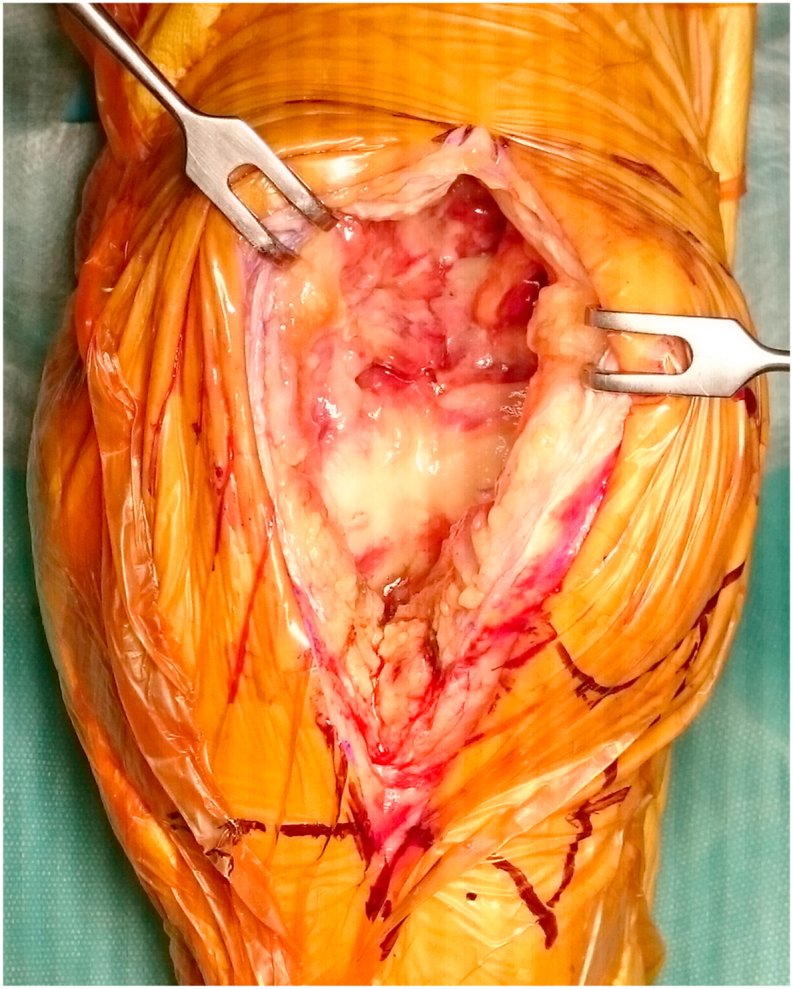
Surgical findings. The synovium was hypertrophic because of inflammatory changes and the suprapatellar sac contained granulation tissues.

The surgical findings were hypertrophic synovium because of inflammatory changes and suprapatellar sac filled with granulation tissues (Fig. 3). We performed the debridement and took more samples for culture examinations. However, the microbiological cultures remained negative at the second surgery.

After the surgical treatment, we started intravenous drip infusion of teicoplanin and oral medical treatment with levofloxacin 500 mg/day and minocycline 200 mg/day considering methicillin-resistant *Staphylococcus aureus*. After starting the antimicrobial therapy, the knee swelling improved and laboratory blood tests also began to improve.

At 4 weeks after the first surgical treatment, intraoperative cultures yielded *Mycobacterium intracellulare*. At this point, we diagnosed knee septic arthritis due to NTM infection. After NTM was detected in the cultures, a three-drug anti-NTM therapy encompassing rifampin, ethambutol, and levofloxacin was started.

A computed tomography (CT) scan of the lungs showed pulmonary ground-glass appearance and inflammatory scars, but sputum cultures and antibody tests for *Mycobacterium avium* complex (MAC) were negative. Therefore, pulmonologists considered that NTM pneumonia was negative.

The patient was maintained on the oral three-drug medical treatment for 1 year. At the follow-up examination after 1 year of treatment, the knee range of motion was improved to 0° of extension and 140° of flexion. There was no evidence of recurrence of the infection by both clinical findings or laboratory data. Repeated arthrocentesis and cultures for mycobacteria yielded no growth.

## 3 Discussion

We found that knee septic arthritis due to NTM infection can present after arthrocentesis for idiopathic hemarthrosis of the knee. We experienced a case of knee septic arthritis due to NTM infection that was successfully treated by surgical debridement and three-drug medical treatment for 1 year.

Most reported musculoskeletal system NTM infections are acquired by direct inoculation after penetrating trauma, surgical procedures, and/or needle injections [3]. Previous reports described arthritis due to NTM infection associated with prosthetic joint infections [6] and intra-articular injections at the clinic [4]. In the latter report, the patients had a history of multiple intra-articular injections of steroids at a local clinic. However, there have been no reports of NTM infections after arthrocentesis for idiopathic hemarthrosis of the knee.

Hematogenous spread of infection from the lungs can also occur. However, hematogenous spread of NTM infections is uncommon [7], and sputum cultures and antibody tests for MAC were negative in our patient. From the above, we suspected that the infection occurred by direct inoculation after an injection.

There is no firmly established standardized treatment regimen for septic arthritis due to NTM infection [8]. The recommended duration of therapy for septic arthritis due to NTM infection is 6–12 months [1], but the duration of treatment depends on multiple factors, including infection site, NTM species, and treatment response. In addition, surgical therapies are often required. In a previous study, the median duration of treatment for bone and joint infections was 1 year [7]. Our patient showed an excellent prognosis with surgical debridement and three-drug medical treatment for 1 year.

Clinically, the diagnosis of septic arthritis due to NTM infection is not easy. The symptoms were reported to include local pain, joint stiffness, swelling, and low-grade fever. However, because the infection often follows a chronic course, the average time from onset of symptoms to diagnosis may be as long as 10 months [3]. Cultures and/or tissue biopsies are necessary for definitive diagnosis of the causative agent [9], and the presence of granulomatous regions in histopathological examinations is useful for diagnosis [10]. Nevertheless, selection of examination depends on clinical suspicion, and thus NTM infections should be considered for patients with elevation of inflammatory markers after episodes of penetrating trauma, surgical procedures, and/or needle injections. In our case, we were able to diagnose NTM infection within 1 month, owing to the consideration of tuberculosis infection at the time of the first surgery.

## 4 Conclusion

We experienced a case of knee septic arthritis due to NTM infection. Because it was possible to diagnose the infection relatively early, it was successfully treated by surgical debridement and three-drug medical treatment for 1 year. Thus, clinicians should consider arthritis due to NTM infection when they encounter patients with incomprehensible swelling and heat after arthrocentesis.

## Please state any sources of funding for your research

None.

## Ethical approval

Research studies involving patients require ethical approval. Please state whether approval has been given, name the relevant ethics committee and the state the reference number for their judgement.

## Author contribution

Please specify the contribution of each author to the paper, e.g. study concept or design, data collection, data analysis or interpretation, writing the paper, others, who have contributed in other ways should be listed as contributors.

## Registration of research studies

In accordance with the Declaration of Helsinki 2013, all research involving human participants has to be registered in a publicly accessible database. Please enter the name of the registry and the unique identifying number (UIN) of your study.

## Provenance and peer review

Not commissioned, externally peer-reviewed.

## Source of funding

The authors have no sponsors.

## Consent

Written informed consent was obtained from the patient for publication of this case report and accompanying images. A copy of the written consent is available for review by the Editor-in-Chief of this journal on request.

## Author contribution

A.M., S.M., M.K., K.Y., and S.A. managed the case and redaction and correction of the manuscript. M.A. and D.T. assisted with redaction, correction, and reconstruction of the manuscript.

## Declaration of competing interest

None.
